# Small intestine remodeling in male Goto–Kakizaki rats

**DOI:** 10.14814/phy2.14755

**Published:** 2021-02-13

**Authors:** Joice Naiara Bertaglia Pereira, Gilson Masahiro Murata, Fabio Takeo Sato, Aline Rosa Marosti, Carla Roberta de Oliveira Carvalho, Rui Curi

**Affiliations:** ^1^ Interdisciplinary Post‐Graduate Program in Health Sciences Cruzeiro do Sul University São Paulo Brazil; ^2^ Department of Medical Clinic Faculty of Medicine University of São Paulo São Paulo Brazil; ^3^ Department of Genetics Evolution, Microbiology and Immunology Institute of Biology State University of Campinas Campinas Brazil; ^4^ Department of Medicine University Center of Maringa Maringá Brazil; ^5^ Department of Physiology and Biophysics Institute of Biomedical Sciences University of São Paulo São Paulo Brazil; ^6^ Butantan Institute São Paulo Brazil

**Keywords:** enteric nervous system, GK rats, inflammation, small intestine morphology, type 2 diabetes

## Abstract

**Background:**

Obesity is associated with the development of insulin resistance (IR) and type‐2 diabetes mellitus (T2DM); however, not all patients with T2DM are obese. The Goto–Kakizaki (GK) rat is an experimental model of spontaneous and non‐obese T2DM. There is evidence that the intestine contributes to IR development in GK animals. This information prompted us to investigate small intestine remodeling in this animal model.

**Methods:**

Four‐month‐old male Wistar (control) and GK rats were utilized for the present study. After removing the small intestine, the duodenum, proximal jejunum, and distal ileum were separated. We then measured villi and muscular and mucosa layer histomorphometry, goblet cells abundance, total myenteric and submucosal neuron populations, and inflammatory marker expression in the small intestinal segments and intestinal transit of both groups of animals.

**Key Results:**

We found that the GK rats exhibited decreased intestinal area (*p* < 0.0001), decreased crypt depth in the duodenum (*p* = 0.01) and ileum (*p* < 0.0001), increased crypt depth in the jejunum (*p* < 0.0001), longer villi in the jejunum and ileum (*p* < 0.0001), thicker villi in the duodenum (*p* < 0.01) and ileum (*p* < 0.0001), thicker muscular layers in the duodenum, jejunum, and ileum (*p* < 0.0001), increased IL‐1β concentrations in the duodenum and jejunum (*p* < 0.05), and increased concentrations of NF‐κB p65 in the duodenum (*p* < 0.01), jejunum and ileum (*p* < 0.05). We observed high IL‐1β reactivity in the muscle layer, myenteric neurons, and glial cells of the experimental group. GK rats also exhibited a significant reduction in submucosal neuron density in the jejunum and ileum, ganglionic hypertrophy in all intestinal segments studied (*p* < 0.0001), and a slower intestinal transit (about 25%) compared to controls.

**Conclusions:**

The development of IR and T2DM in GK rats is associated with small intestine remodeling that includes marked alterations in small intestine morphology, local inflammation, and reduced intestinal transit.

## Graphical abstract

Goto–Kakizaki (GK) rat is an experimental model of spontaneous and non‐obese type‐2 diabetes mellitus—T2DM).GK rats exhibit decreased small intestinal area, increased crypt depth, villi height and thickness, and muscular layer thickness, increased content of IL‐1β and NF‐κB p65, decreased density of submucosal neurons, myenteric and submucosal neuronal and ganglionic hypertrophy, and decreased intestinal transit.The development of IR and T2DM in GK rats is associated with small intestine remodeling with marked changes in morphology, enteric nervous system, and transit.

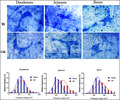

## 1 INTRODUCTION

Insulin resistance (IR) state is a harmful consequence of obesity and is associated with glucose intolerance, dyslipidemias, increased risk of nonalcoholic hepatic steatosis, atherosclerosis, hypertension, and low‐grade systemic inflammation (Donath & Shoelson, 2011; Shoelson & Goldfine, 2009; Tanti & Jager, 2009). Previous work has shown that the primary sites of obesity‐mediated inflammation are the adipose tissue and liver (Syrenicz et al., 2006; Xu et al., 2003).

Additionally, other studies have demonstrated that intestinal inflammation and intestinal microbiota composition are also involved in obesity and IR development (Garidou et al., 2015; Gil‐Cardoso et al., 2017; Monteiro‐Sepulveda et al., 2015). This observation is not entirely surprising given that the gastrointestinal (GI) tract is responsible for transporting luminal contents, secreting hormones and cytokines, and absorbing ions, water, and nutrients. It has also been shown that the intestinal epithelium participates in pathogen defense and the elimination of harmful compounds (Worthington, 2015).

Ding et al. (2010) described the intestinal inflammation and pro‐inflammatory signaling in multiple intestinal cell types in response to a high‐fat diet. Interestingly, obese humans on a low‐calorie diet exhibited reduced expression of pro‐inflammatory genes, including TNF‐α, IL‐1β, and IL‐8, in the rectosigmoid mucosal, which were associated with weight loss (Pendyala et al., 2011).

In addition to obesity, other inflammatory conditions such as periodontal disease, obstructive pulmonary disease, arthritis, and muscular dystrophy are also associated with the development of IR and type 2 diabetes mellitus (T2DM; Demmer et al., 2008; McNeely & Boyko, 2004; Tiengo et al., 2008). Notably, most studies involving this T2DM and IR were performed using obese humans or animals fed a high‐calorie diet that resembles the western human diet (Lowette et al., 2016; Masi et al., 2017; Nyavor et al., 2020). However, not all patients with T2DM are obese. In Asia, for example, obesity rates are low, and about 60% of T2DM patients are classified as lean (Brunetti, 2007). This observation highlights the need for studies investigating T2DM development in non‐obese humans and animals.

One such model is the Goto–Kakizaki (GK) rat developed by Goto, Kakizaki, and Masaki in 1975. This animal model was obtained by selective reproduction of non‐diabetic Wistar rats with slight glucose intolerance. Consequently, the rats spontaneously develop T2DM without becoming obese (Goto et al., 1975). GK rats exhibit marked age‐dependent metabolic changes in the liver, skeletal muscle, and adipose tissue. They also exhibit moderate hyperglycemia, impaired glucose‐induced insulin secretion, glucose intolerance, peripheral IR, and chronic inflammation (Bisbis et al., 1993; Ostenson et al., 1993; Ouyang et al., 2019). It has been reported that at 4 weeks of age, GK rats have elevated plasma glucose concentrations that reach a hyperglycemic plateau at between 12 and 16 weeks, and high plasma insulin concentrations were observed at 8 weeks of age (Xue et al., 2011).

Previous studies have shown that inflammatory marker expression is increased in various cells and tissues of GK rats, including leukocytes, liver, skeletal muscle, endothelium, and pancreatic β‐cells (Nie et al., 2011; Xue et al., 2015). As alluded to previously, although GK animals exhibit chronic inflammation and IR, they do not accumulate body fat (Xue et al., 2011). In Wistar rats, fat deposit mass increases linearly with age. However, fat deposit mass in GK rats stop increasing between 8 and 12 weeks of age and decreases frequently after week 16 (Xue et al., 2011). Thus, this animal model allows researchers to investigate the development of T2DM without inducing obesity and/or utilizing a high‐calorie diet.

It has been shown that the gut microbiota composition in the GI tract and fecal metabolic phenotype of GK rats are markedly different from Wistar rats (Peng et al., 2020). Moreover, several GI‐related characteristics such as small intestine hyperplasia, augmented disaccharidase activity, increased intestinal area, and small intestinal dysrhythmias were reported in GK animals (Adachi et al., 2003; Ouyang et al., 2015; Zhao et al., 2003). Furthermore, changes in the morphology of the small intestine can lead to biomechanical alterations, including impaired intestinal sensory function and intestinal motility (Zhao et al., 2003, 2006; Zoubi et al., 1995).

Previously, Salinari et al. (2014) investigated the role of the small intestine in the regulation of insulin sensitivity in GK rats. The authors found that both surgical resection and proximal small intestine bypass improved insulin sensitivity through an incretin‐independent mechanism. Based on these results, it was concluded that the small intestine is involved in the development of IR and T2DM in the GK rat animal model.

Since diabetic patients commonly present impaired GI tracts with morphological changes that impair absorption and motility, we sought to monitored small intestine remodeling and motility in GK rats. To achieve this goal, we removed the small intestines, separated the duodenum, proximal jejunum, and distal ileum, and analyzed the histomorphometry of the villi and muscular and mucosa layers, goblet cell abundance, the total myenteric and submucosal neuron populations, and inflammatory marker expression. Additionally, we evaluated the intestinal transit time in Wistar and GK rats.

## 2 MATERIALS AND METHODS

### 2.1 Animals

We obtained the Wistar and GK rats from Charles River Laboratories International Inc. All animals were housed in the Interdisciplinary Post‐graduate Program in Health Sciences animal facility at Cruzeiro do Sul University, where they were maintained at 23 ± 2°C, with a light/dark cycle of 12 h (lights on at 6:30 AM). Both groups of rats had free access to standard rodent chow (Nuvilab®) and water until 16 weeks of age.

**FIGURE 1:**
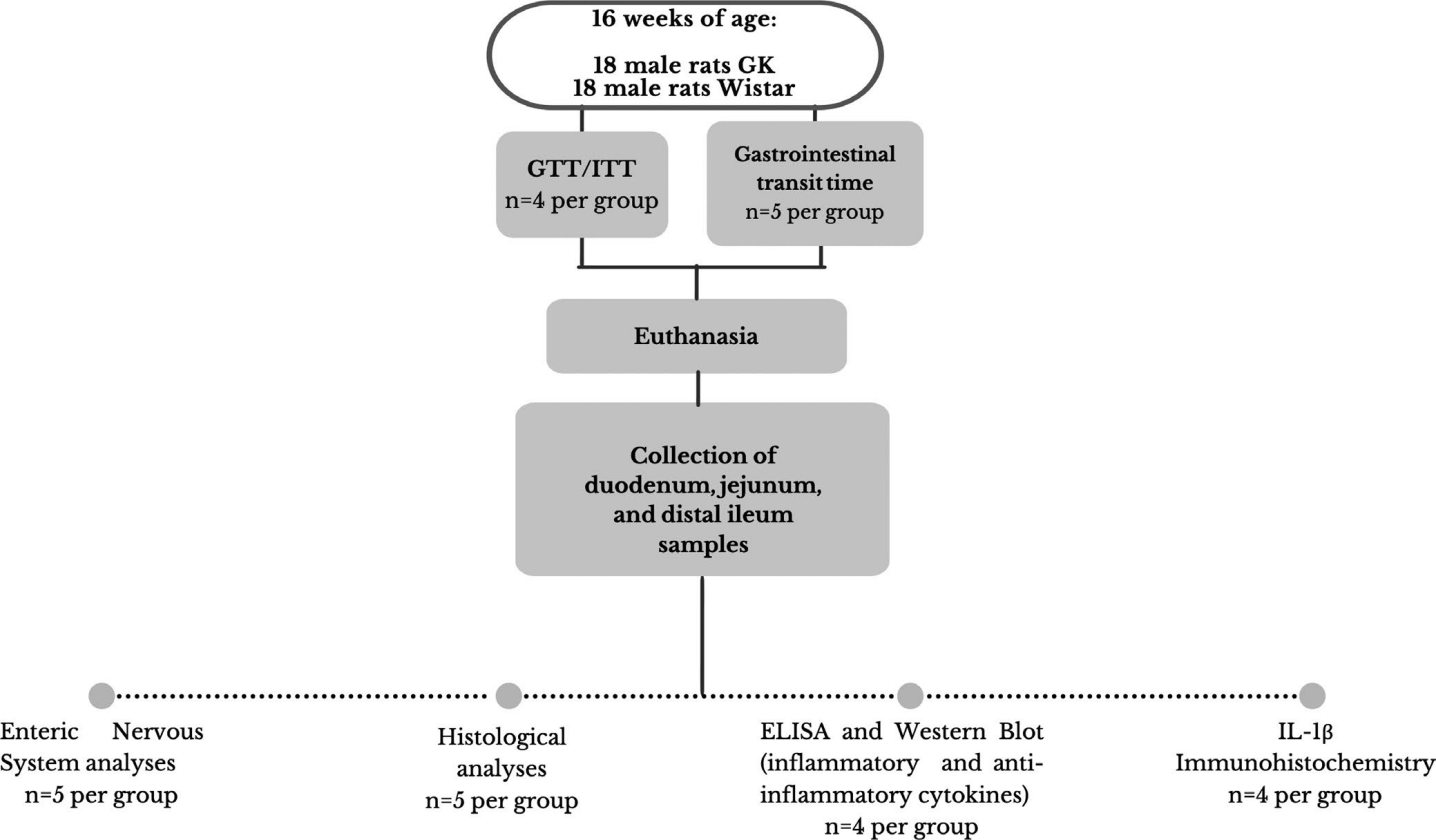
Schematic representation of the animal groups (Wistar and GK), experimental protocol and analyses. Thirty‐six male rats were randomly allocated into two groups (*n* = 18 rats/group): control (Wistar) and experimental (GK). Four animals from each group were randomly selected to undergo a glucose tolerance test (GTT). Four days after the GTT, the same animals were subjected to an insulin tolerance test (ITT). Ten animals (five per group) were randomly selected for the GI transit measurement. At 16 weeks of age, all 36 control and experimental animals (18 per group) were weighed, euthanized by CO_2_ inhalation, and decapitated. Following euthanasia, the duodenum, jejunum, and distal ileum were collected and processed for enteric nervous system, histological, ELISA, western blot, and immunohistochemistry analyses

Thirty‐six male rats were randomly allocated into two groups (*n* = 18 rats/group): control (Wistar) and experimental (GK). The maximum caging density was three animals of the same group. All efforts, including a pilot study that allowed us to standardize all the procedures, were made to minimize the number of animals used and their suffering. The experimental procedures (Figure 1) were approved by the Animal Ethical Committee at the Cruzeiro do Sul University (protocol number 024/2017).

### 2.2 Glucose tolerance test

Ten days before euthanasia, four animals from each group were randomly selected to undergo a glucose tolerance test (GTT). Briefly, after 12 h of fasting, the animals were given intraperitoneal (IP) injections of 50% aqueous glucose solution (2 g per kg). A drop of blood obtained via a tail tip cut was collected onto a test strip, and blood glucose concentrations were measured with a blood glucose monitor (AccuCheck). Samples were taken at the following time points: 0 (before the glucose injection) and 15, 30, 60, and 90 min after the injection. The area under the curve was then calculated by using the integrated trapezoid rule (Matthews et al., 1990).

### 2.3 Insulin tolerance test

Four days after the GTT, the same animals were subjected to an insulin tolerance test (ITT). For this test, rats were given IP injections of insulin (Novolin, Novo Nordisk A/S, Copenhagen, Denmark) at the dose of 0.75 IU per kg, after 12 h fasting. Blood samples were obtained as described above and measured at the following time points: 0 (before the insulin injection) and 4, 8, 12, 20, 30, and 40 min after the insulin injection (Kuwabara et al., 2017). The area under the curve was also calculated as previously described for the GTT.

### 2.4 Total GI transit measurement

Ten days before euthanasia, ten animals (five per group) were randomly selected for the GI transit measurement. Rats were administered 300 μl of 2.5% methylene blue (Mendes et al., 2015) associated with methylcellulose dissolved in water by gavage and housed in individual cages. The time that elapsed from the moment of administration to the elimination of the first blue feces pellet was recorded.

### 2.5 Body mass, nose‐to‐tail length, and adipose tissue weight

At 16 weeks of age, after 8 h of fasting, all 36 control and experimental animals (18 per group) were weighed, the nose‐to‐tail length was measured using a metric tape measure on a clean laboratory bench, and then euthanized by CO_2_ inhalation, and decapitated. The retroperitoneal, epididymal, and mesenteric adipose tissues were harvested and weighed. The adipose tissues were normalized according to the nose‐to‐tail length of each animal (Kuwabara et al., 2017).

### 2.6 Histological preparation of the tissue samples

A total of 10 animals (five per group) were used for the histological analyses. Small intestine segments including the duodenum, proximal jejunum, and distal ileum were collected (within the anatomical limits, see below), fixed in buffered formalin, dehydrated, diaphanized in xylene, and embedded in paraffin. The anatomical limits that separated each intestinal segment included the duodenojejunal flexure (duodenum from jejunum), the ileocecal fold (cranial limit), and the ileocecal junction—caudal limit (distal ileum). Five semi‐serial 4 μm sections were obtained from the paraffin‐embedded intestinal segments. Each section was stained with hematoxylin and eosin (HE) for the descriptive and morphometric analyses and with Periodic Acid‐Schiff (PAS), counterstained with hematoxylin, for the identification and quantification of the mucosecretory cells (goblet cells).

### 2.7 Histological analyses

Using images captured with a camera coupled to a trinocular microscope (Axio Cam ERc 5S, Axiovert 40; Zeiss), a blinded observer measured the crypt depth, villi height and thickness, muscular layer thickness, and reported the presence of inflammatory infiltrate in the small intestine. For the measurements, four fields from each of the five histological sections for each animal were photographed. Each histological section was divided into quadrants, and images from four different areas from the mesenteric insertion were captured. Crypt depth, villi height and thickness, and muscular layer thickness were calculated based on 20 random measurements per animal in each HE stained segment. The quantification of goblet cell abundance was performed as previously described (Góis et al., 2016). Briefly, 2500 consecutive cells, including PAS positive or negative, were counted in each specimen. The percentage of the goblet cells was calculated by dividing the number of PAS‐positive cells by the total number of quantified cells. All measurements were performed using the Axio Vision Rel. 4.5 software (Zeiss). The same images used for the morphometric analyses (HE stained sections) were also used to qualitatively determine the amount of intestinal polymorphonuclear leukocytes (neutrophils) present in the samples.

### 2.8 Measurements of inflammatory and anti‐inflammatory cytokines by ELISA

Eight animals (four per group) were used to measure cytokine concentrations. Following euthanasia, the duodenum, jejunum, and distal ileum were frozen and then pulverized in liquid nitrogen. About 100 mg of each tissue segment was homogenized in Tris‐EDTA buffer containing 1% SDS (sodium dodecyl sulfate) and a protease and phosphatase inhibitor cocktail (Sigma Aldrich). The samples were centrifuged at 1000 *g*, for 10 min at 4°C, and the supernatant was collected. The concentrations of the pro‐inflammatory cytokines tumor necrosis factor‐α (TNF‐α), interleukin‐6 (IL‐6), and IL‐1β, and the anti‐inflammatory cytokine IL‐10 were measured using DuoSet ELISA Development System kits (R&D Systems), according to the manufacturer's instructions. The detection limits of the ELISA kits used are 62.4–4000 pg/ml for TNF‐α, 122–8000 pg/ml for IL‐6, and 62.5–4000 pg/ml for IL‐1β and IL‐10.

### 2.9 Tissue localization of IL‐1β by immunohistochemistry

A total of eight animals (four per group) were used for the immunohistochemistry analyses. The small intestine segments were transferred to Tissue‐Tek® medium (Sakura Finetek), frozen in liquid nitrogen, and sliced into 6‐μm‐thick sections. We performed the immunohistochemistry technique using the Mouse and Rabbit Specific HRP/DAB IHC Micro‐polymer detection kit (ab236466, Abcam), following the manufacturer's instructions. The slides were blocked with 3% BSA and incubated overnight with rabbit anti‐IL‐1β antibody (ab9722, Abcam) diluted 1:250. The antigen–antibody complex was detected using chromogen 3,3′‐diaminobenzidine (DAB). Sections without the primary antibody (IL‐1β) were used as a negative control for the immunolabeling process. This qualitative evaluation was employed to identify the enteric immunoreactive cells. Photomicrographs were captured with an Eclipse® microscope (Nikon) equipped with a 40× objective.

### 2.10 Western blot analysis

The previously processed small intestine segments (duodenum, proximal jejunum, and distal ileum) used for the ELISA experiments were also used for Western blotting analysis (four per group). For this purpose, the samples were homogenized, and the total protein content was determined as previously described (Kuwabara et al., 2017). Equal amounts of total protein (10 μg) were then separated by SDS‐PAGE and transferred to nitrocellulose/PVDF membranes. The total protein loading for each sample was normalized by Ponceau S staining (Fortes et al., 2016). Previous studies reported the appropriateness of this procedure for protein loading quantitation (Fortes et al., 2016). The images were captured using an Amersham Imager 600 (Amersham/GE Healthcare) and quantified using the Image J software (NIH). The primary antibody used (dilution 1:1000) was NF‐κB P65 from Cell Signaling Technology (Danvers).

### 2.11 Intestine length and serosal surface area

Immediately after removal, the length of the small intestine was determined using a metric tape measure on a clean laboratory bench to prevent stretching. Afterward, to avoid the retraction of the organ at the moment of the image capturing, the intestine was placed between a sheet of graph paper and a thin glass plate and photographed. The serosal surface area (cm^2^) was determined by analyzing the width and length of the small intestine using the Axio Vision Rel. 4.5 software (Da Silva et al., 2012). A total of 10 animals (five per group) were used for this set of experiments.

### 2.12 Whole‐mount preparations

We washed nearly 3 cm of each small intestine segment with saline and tied up one of the ends. Using a syringe, we injected the Giemsa fixative solution (acetic formalin) into the intestinal lumen through the free end; the volume injected promoted a slight distension of the intestine wall similar to that produced by an alimentary bolus. The intestine free end was tied, and the viscera was immersed in the Giemsa's fixative solution for 48 h. Next, we dissected the intestinal segments using a stereomicroscope to obtain whole mounts of the myenteric and submucosal plexuses. The myenteric plexus was obtained by removing the submucosal tissue and mucous layer. The submucosal plexus was obtained by removing the mucosal and muscular layer (Trevizan et al., 2019). A total of 10 animals (five per group) were used for this set of experiments.

### 2.13 Morphoquantitative analysis of myenteric and submucosal plexuses

The whole mounts of the myenteric and submucosal plexuses were stained with Giemsa's solution, and the slides were mounted with glycerin in PBS (1:2). The enteric neurons are stained due to the high affinity of the Nissl bodies to methylene blue dye (Giemsa's dye). The enteric neurons are differentiated from other cell types by intense cytoplasmic staining (Barbosa, 1978).

Sixty images of the whole‐mount preparations were randomly captured, and we used a test system with inclusion and exclusion lines (100 cm^2^) for the neuronal quantification per unit area. The neurons within the test area boundaries and those that touched the inclusion lines were counted, whereas the neurons that touched exclusion lines were not. Myenteric and submucosal neuronal densities were expressed as the number of cells per mm^2^. We estimated the total number of myenteric and submucosal neurons in the small intestine by multiplying the number of cells/mm^2^ by the previously determined serosal surface area. We measured the areas of 100 neuronal cell bodies (μm^2^) per segment per animal using a semi‐automatic morphometric device (Pereira et al., 2014), totaling 500 neurons in each intestinal segment per group.

We randomly captured images of 40 ganglia per segment per group of each plexus and then quantified the number of myenteric and submucosal neurons per ganglion and measured the ganglion area.

### 2.14 Statistical analysis

We used two‐way ANOVA and multiple comparisons by Sidak's test to analyze GTT, and ITT time points repeated‐measure–based parameters (time and phenotype). Results are expressed as the mean ± SEM.

We first tested the data for a normal distribution for each parameter assessed. We compared the group data for each intestinal segment using an unpaired Student's *t*‐test or the equivalent nonparametric Mann–Whitney U test using the Prism 8.4.3 software (GraphPad). Results are expressed as the mean ± SEM and compared using the Student's *t*‐test or as median percentiles (25%; 75%) compared using the Mann–Whitney test. The *p* values less than or equal to 0.05 were considered statistically significant.

## 3 RESULTS

### 3.1 Body mass, nose‐to‐tail length, and adipose tissues weight

**FIGURE 2:**
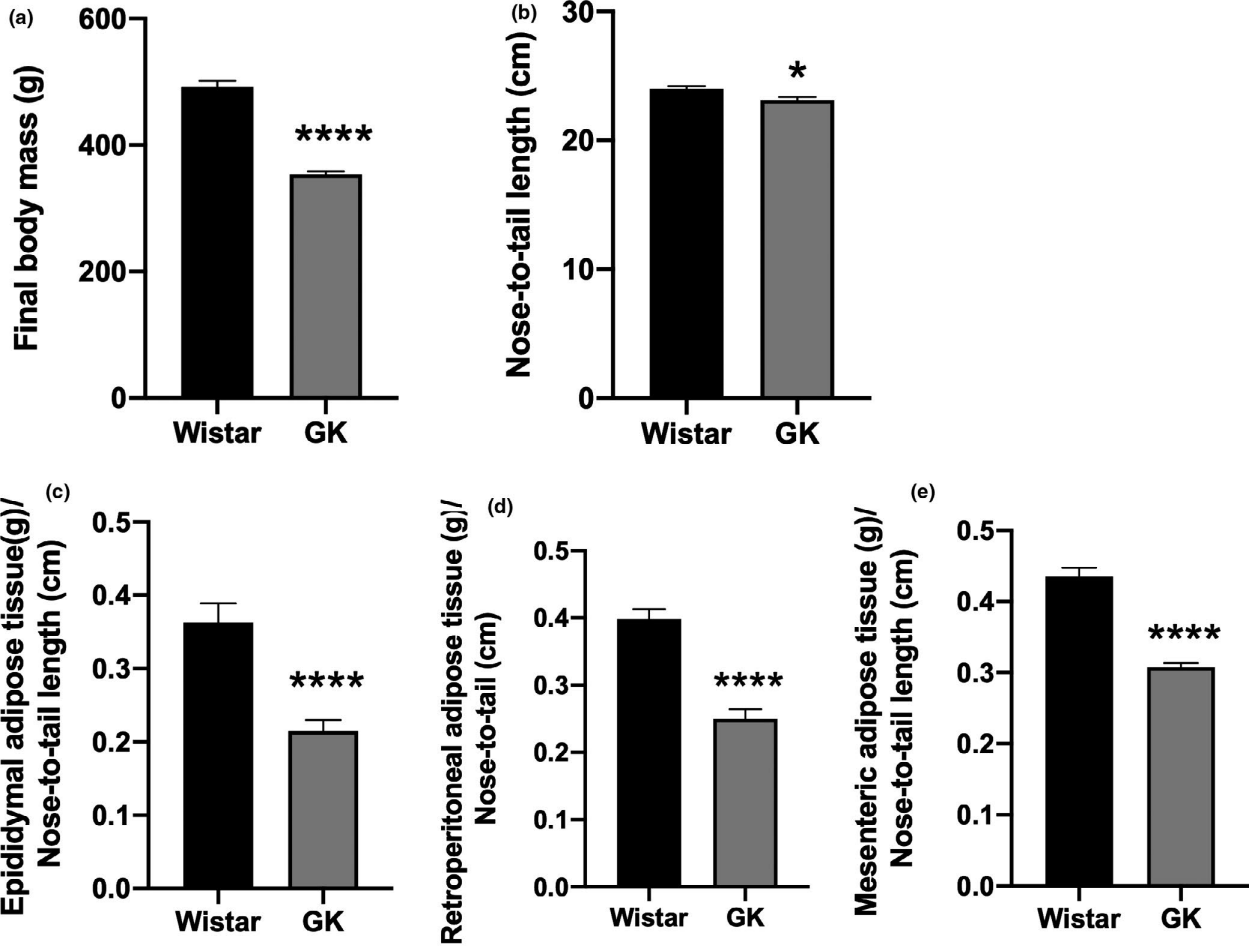
(a) Body mass (Wistar 472 ± 10.7 versus GK 369 ± 3.0) and (b) nose‐to‐tail length (Wistar 24 ± 0.2 vs GK 23 ± 0.2). (c) Epididymal (Wistar 0.36 ± 0.02 vs GK 0.21 ± 0.01), (d) retroperitoneal (Wistar 0.39 ± 0.01 vs GK 0.24 ± 0.01), and (e) mesenteric (Wistar 0.43 ± 0.01 vs GK 0.30 ± 0.005) adipose tissues weights of the Wistar and GK groups at 16 weeks old. Adipose tissues (g) were normalized based on the nose‐to‐tail length (cm). *p < 0.05; **p < 0.01; ****p < 0.0001

At 16 weeks of age, the body mass of the GK group (369.1 ± 3.0 g) was 21% lower than that of the Wistar rats (472 ± 10.7 g) (*p *< 0.0001). Additionally, the GK group exhibited a significantly reduced nose‐to‐tail length (*p* < 0.05) and lower retroperitoneal (*p* < 0.0001), epididymal (*p* < 0.0001), and mesenteric (*p* < 0.0001) adipose tissue weights compared to the control Wistar rats (Figure 2).

### 3.2 Glucose and insulin tolerance tests

**FIGURE 3:**
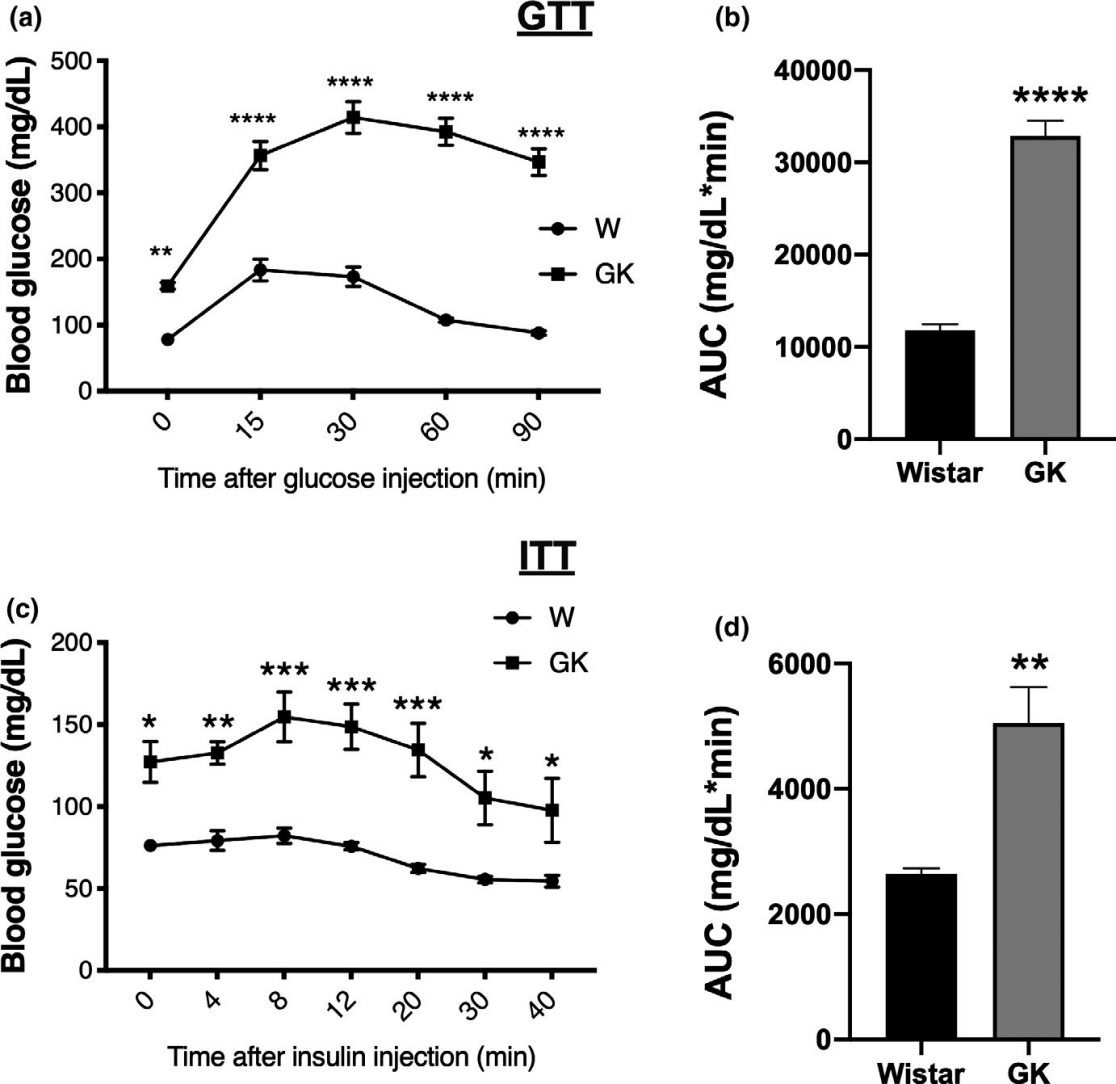
(a) Blood glucose concentrations measured before (time 0) and during the glucose tolerance test—GTT. (b) GTT area under the blood glucose concentration curve (Wistar 11,783 ± 666 vs GK 32850 ± 1651). (c) Blood glucose concentrations measured before (time 0) and during the insulin tolerance test—ITT. (d) ITT area under the blood glucose concentrations curve (Wistar 2641 ± 90.7 vs GK 5049 ± 576.1). *p < 0.05; **p < 0.01; ***p < 0.001; ****p < 0.0001

The GK group presented elevated fasting blood glucose concentrations compared to the Wistar group. As shown in Figure 3a, following glucose loading, the control rats reached the glycemic peak after 15 min, after which the glucose blood concentrations gradually decreased until reaching the values observed in the fasting condition. In contrast, blood glucose concentrations of the GK rats gradually increased for 30 min (peak) and remained elevated until the end of the experimental period (90 min) (Figure 3a). The area under the GTT curve for the GK group (32,850 ± 1651) was nearly threefold higher than the Wistar group (11,783 ± 666) (*p* < 0.0001) (Figure 3b).

In the ITT, the glucose concentration of the GK group remained elevated until 8 min after the insulin injection. After 30 min, the glucose concentrations of the GK animals reached the fasting condition values of the Wistar rats (Figure 3c). The area under the ITT curve for the GK group (5049 ± 576.1) was significantly greater (*p* = 0.006) than that of the Wistar group (2641 ± 90.7) (Figure 3d).

### 3.3 Small intestine length

There was no significant difference in the small intestine length between groups (Table 2). Although the nose‐to‐tail length of the GK rats was about 4% shorter (*p* < 0.05), when we normalized the intestine length by the nose‐to‐tail length, we did not find any significant differences (data not shown).

### 3.4 Morphometric analyses

The intestinal morphometric analyses revealed that the intestinal sections of the GK rats were significantly thicker than the Wistar rats. For example, the thickness of the duodenum was increased by 19% (*p* < 0.0001), the jejunum by 32% (*p* < 0.0001), and the ileum by 64% (*p* < 0.0001). Additionally, the GK rats exhibited decreased crypt depth in the duodenum (9%, *p* < 0.05) and ileum (20%, *p* < 0.0001) and increased crypt depth in the jejunum (17%, *p* < 0.0001). Furthermore, the GK group's villi height was 54% higher in the jejunum, and 21% in the ileum (*p* < 0.0001) and the villi thickness was decreased by 11% in the duodenum (*p* < 0.05) and increased by 25% in the ileum (*p* < 0.0001). A complete summary of the morphometric analyses is presented in Table 1.

### 3.5 Goblet cells abundance

As shown in Table 1, there were no significant differences in the percentage of goblet cells present in any small intestine segments when comparing the Wistar and GK groups.

**TABLE 1:** Measurements in the duodenum, jejunum, and ileum of the Wistar and GK groups

	Duodenum		Jejunum		Ileum	
	Wistar	GK	Wistar	GK	Wistar	GK
Villi height (μm)	441 ± 9.9	441 ± 8.6	343 ± 9.5	531 ± 13.4****	308 ± 10.1	375 ± 9.7****
Villi thickness (μm)	114 ± 3.9	101 ± 2.1*	97 ± 3.0	104 ± 3.7	77 ± 2.1	97 ± 2.9****
Crypt depth (μm)	251 ± 6.0	228 ± 3.1*	192 ± 4.1	224 ± 5.1****	247 ± 3.8	196 ± 3.8****
Muscle thickness (μm)	109 ± 2.8	130 ± 4.1****	90 ± 2.6	119 ± 3.7****	86 ± 2.3	141 ± 4.6****
Goblet cells (%)	16 ± 0.4	16 ± 1.0	25 ± 0.8	25 ± 0.2	31 ± 0.6	31 ± 0.2

Results are expressed as the mean ± SEM and comparisons were made using Student's *t*‐test.

* *p* < 0.05;

**** *p* < 0.0001.

### 3.6 Intestine polymorphonuclear leukocytes

Qualitative analyses revealed no difference in the amount of infiltrating inflammatory cells present in the small intestines of the Wistar and GK groups.

### 3.7 Intestinal cytokine concentrations

**FIGURE 4:**
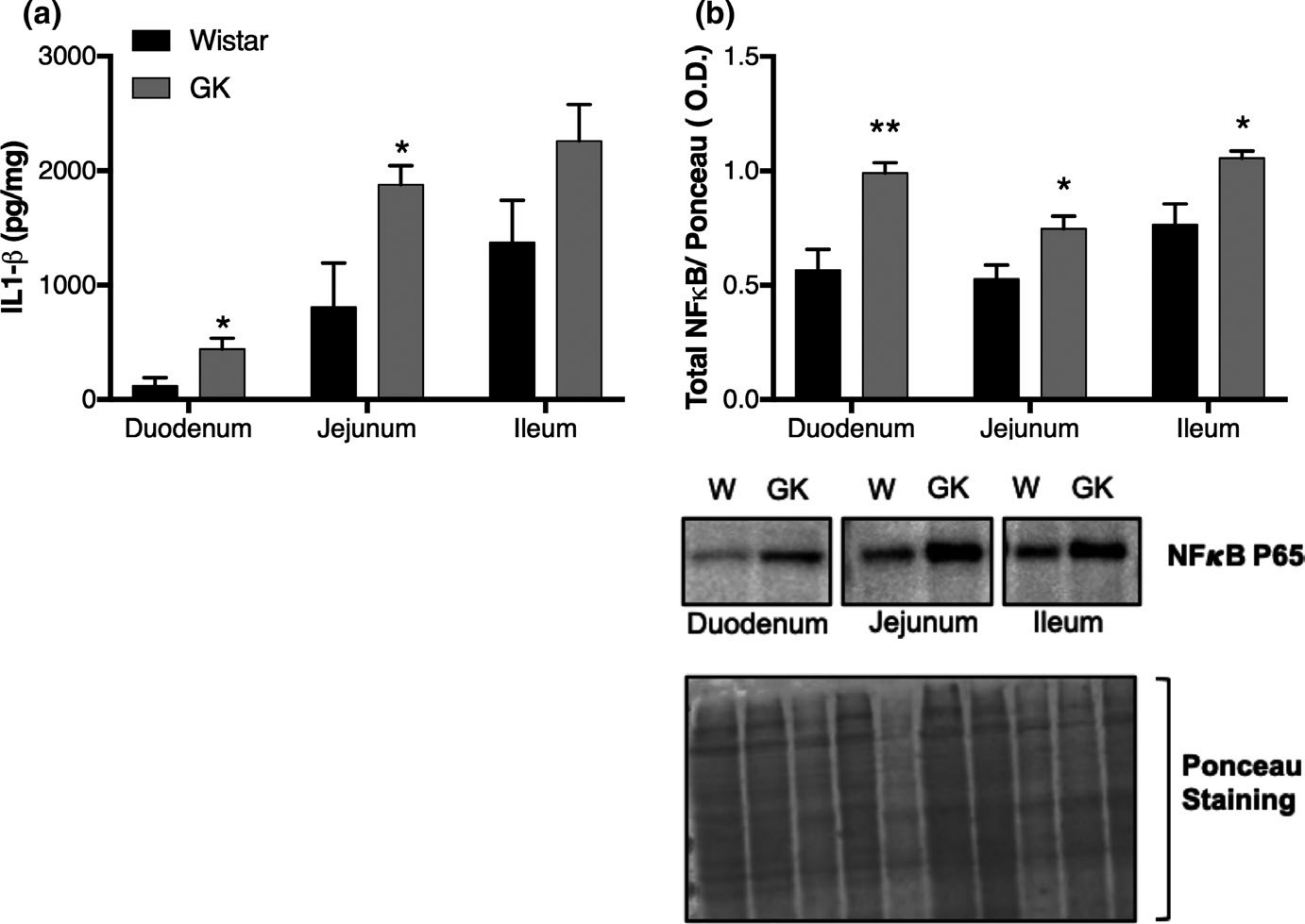
(a) Interleukin‐1β concentrations in the small intestine (duodenum, jejunum, and ileum) of the GK and Wistar groups as determined by ELISA. (b) Total NFκB P65 content in the small intestine (duodenum, jejunum, and ileum) as determined by western blotting. Graphs present the results of the band intensities. We included representative membranes of the NF‐kB P65 bands and Ponceau staining. *p < 0.05; **p < 0.01

Compared to the control group, the small intestine of the GK rats had an increased IL‐1β concentrations (pg/mg) in the duodenum (441.2 ± 95.5 vs 120.6 ± 70.1, *p* < 0.05) and jejunum (1877 ± 165.9 vs 805.9 ± 387.1, *p* < 0.05). While elevated concentrations of this cytokine were detected in the ileum of GK rats (2260.0 ± 318.5 vs 1371 ± 370.9, *p* = 0.1), this increase failed to reach a level of statistical significance (Figure 4a). Concerning IL‐6, TNF‐α, and IL‐10 content, we were unable to detect a significant difference between groups, or the measured levels fell below the detection limit of the ELISA kits (data not shown).

Additionally, we observed an increase in total NF‐kB p65 content (O.D.) in the duodenum (0.99 ± 0.04, *p* < 0.01), jejunum (0.74 ± 0.05, *p* < 0.05), and ileum (1.05 ± 0.03, *p* < 0.05) of GK rats when compared to the Wistar group (duodenum 0.56 ± 0.09, jejunum 0.52.±0.06, and ileum 0.76 ± 0.09) (Figure 4b).

### 3.8 IL‐1β localization by immunohistochemistry

**FIGURE 5:**
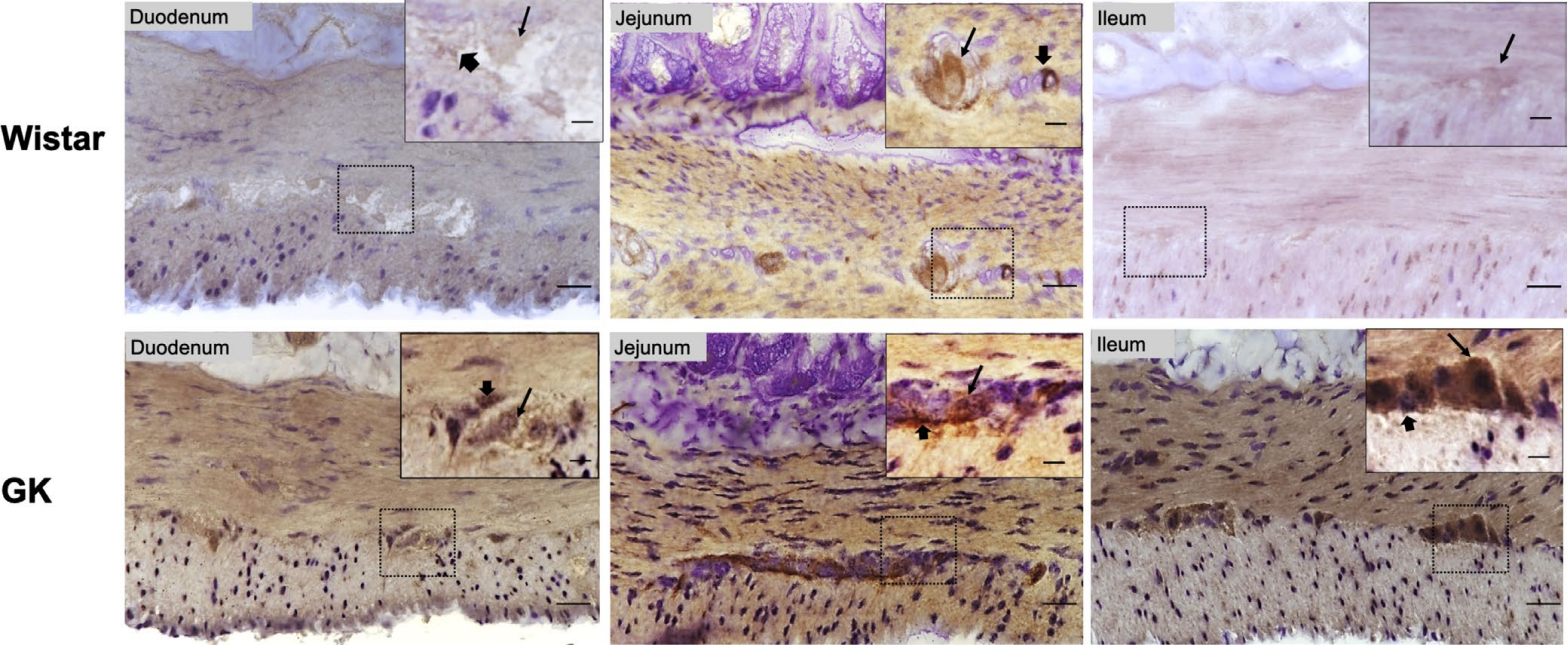
Representative photomicrographs of IL‐1β immunohistochemistry in the duodenum, jejunum, and ileum of the GK and Wistar groups. Thin arrows indicate IL‐1β immunoreactive myenteric neurons and thick arrows indicate IL‐1β immunoreactive glial cells. Objective: Bottom image 40×, Top image 60×. Scale bars: Bottom image 20 μm, Top image 15 μm

The results reported in the ELISA were confirmed using the qualitative intestinal IL‐1β immunohistochemistry assay. In the Wistar animals, we observed low IL‐1β reactivity in the muscle layer, myenteric neurons, and glial cells of the duodenum, jejunum, and ileum. This result was in stark contrast to the GK rats, which exhibited intense staining in the muscle layer, myenteric neurons, and glial cells in all intestinal segments (Figure 5).

### 3.9 Myenteric plexus analyses

The neuronal ganglion density was not different in any of the small intestine segments when comparing the Wistar and GK groups. However, the myenteric ganglion area increased by 37% (*p* < 0.05) in the duodenum, 33% (*p* < 0.0001) in the jejunum, and 32% (*p* < 0.0001) in the ileum of GK rats (Table 3).

The number of neurons per mm^2^ in each small intestine segment was statistically equivalent in both groups of animals (Table 3). However, the serosal surface area of the small intestine was significantly reduced in the GK animals. Moreover, the total number of small intestine neurons was attenuated in the GK group (Table 2).

**TABLE 2:** Measurements in the small intestine of the Wistar and GK groups

Parameter	Wistar	GK
Intestine length (cm)	101 ± 5.1	103 ± 1.1
Serosal surface area (cm^2^)	157 ± 3.0	105 ± 3.6****
Estimated total number of myenteric neurons/mm^2^ (×10^6^)	7.4 ± 0.4	5.0 ± 0.3****
Estimated total number of submucosal neurons/mm^2^ (×10^6^)	4.4 ± 0.1	2.7 ± 0.07****
Intestinal transit time (min)	541 ± 17.7	715 ± 14.8**

Results are expressed as the mean ± SEM and comparisons were made using Student's *t*‐test.

** *p* < 0.01;

**** *p* < 0.0001.

**FIGURE 6:**
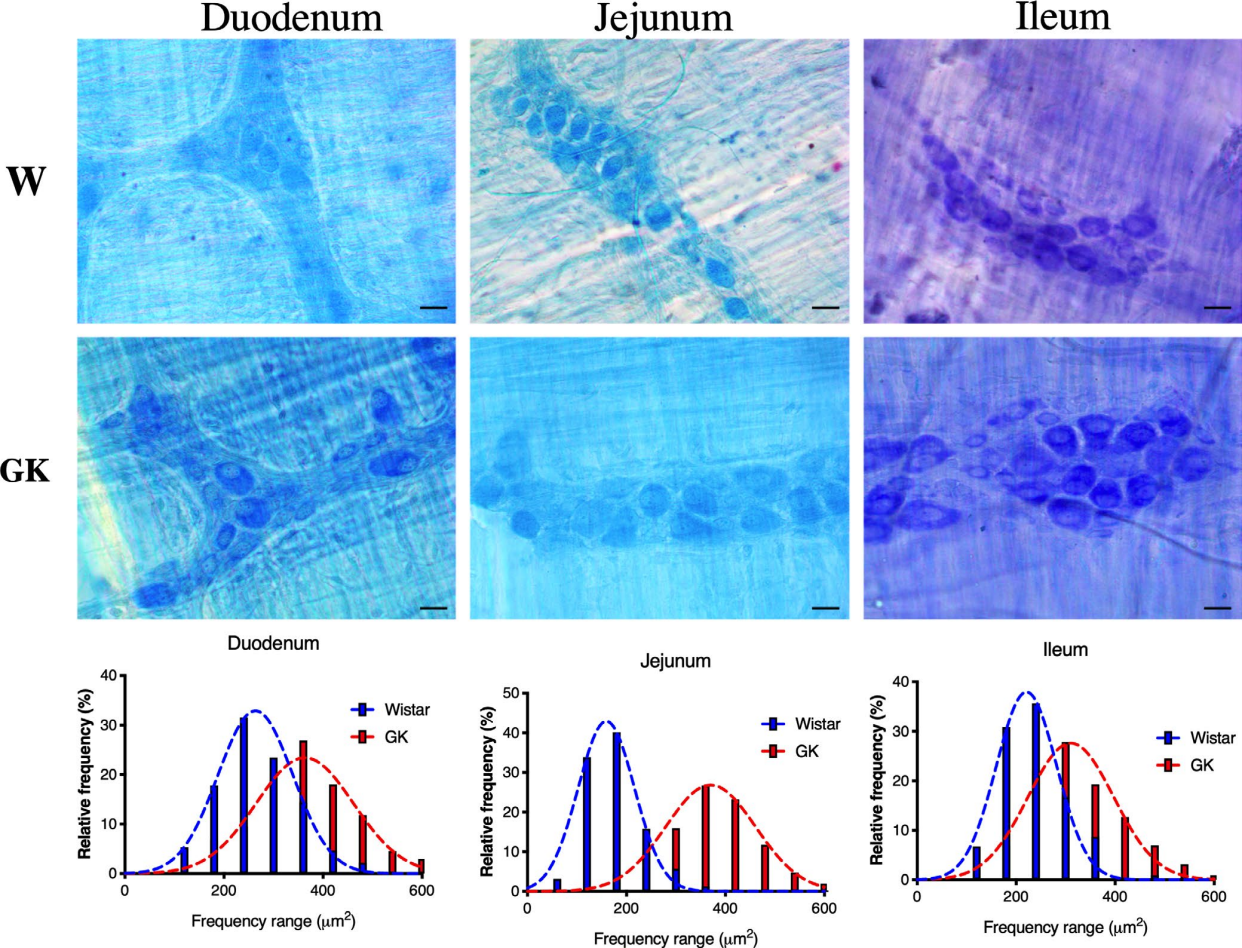
Representative photomicrographs of the myenteric neurons and frequency histogram related to the neuronal areas in the duodenum, jejunum, and ileum of the Wistar and GK groups are indicated. Objective: 40×, Scale bars: 20 μm

The neuronal body area was increased (*p* < 0.0001) by 34% in the duodenum, 111% in the jejunum, and 40% in the ileum of the GK rats. In the duodenum, neuron size ranged from 240 to 300 μm^2^ in the Wistar group and from 360 to 420 μm^2^ in GK rats. The neuronal body area of the jejunum ranged from 120 to 180 μm^2^ in Wistar rats and from 360 to 420 μm^2^ in the GK group. In the ileum, the dimensions of the myenteric neurons ranged in size from 180 to 240 μm^2^ in the Wistar group and from 300 to 360 μm^2^ in GK rats (Table 3 and Figure 6).

**TABLE 3:** Morphoquantitative analyses of the total myenteric neuronal population in the duodenum, jejunum, and ileum of the Wistar and GK groups

	Duodenum		Jejunum		Ileum	
	Ileum	GK	Wistar	GK	Wistar	GK
Myenteric neuronal density (mm^2^)^a^	492 ± 23	480 ± 13	483 ± 16	496 ± 19	435 ± 7	438 ± 40
Neuronal body area (μm^2^)^b^	265 (215; 326)	365**** (300; 428)	165 (135; 207)	365**** (302; 423)	228 (197; 276)	320**** (265; 400)
Myenteric neuronal density per ganglion^a^	23 ± 0.7	24 ± 0.7	23 ± 1.0	20 ± 0.9	19 ± 0.5	20 ± 0.5
Myenteric ganglion area (μm^2^)^a^	11,612 ± 495	15,926 ± 396*	11,410 ± 409	15,227 ± 510****	11,047 ± 339	14,590 ± 374****

^a^ Data are expressed as the mean ± SEM and comparisons were made using Student's *t*‐test.

^b^ Data are expressed as median percentiles (25%; 75%) and compared using the Mann–Whitney test.

* *p* < 0.05;

**** *p* < 0.0001.

### 3.10 Submucosal plexus analyses

A reduced number of submucosal ganglion neurons were present in the jejunum (33%, *p* < 0.0001) and ileum (28%, *p* < 0.0001) of GK animals. Moreover, the submucosal ganglion area of the GK rats was increased by 9.5% in the duodenum (*p* < 0.05) and 19% in the ileum (*p* < 0.05) compared to the Wistar group (Table 4).

**TABLE 4:** Morphoquantitative analyses of the submucosal total neuronal population of the duodenum, jejunum, and ileum of the Wistar and GK groups

	Duodenum		Jejunum		Ileum	
	Wistar	GK	Wistar	GK	Wistar	GK
Submucosal neuronal density/mm^2^ ^a^	278 ± 10	290 ± 14	287 ± 13	249 ± 14	300 ± 12	244 ± 4.3**
Neuronal body area (μm^2^)^b^	242 (193; 293)	268*** (203; 312)	217 (173; 271)	367**** (289; 470)	274 (226; 334)	347**** (286; 427)
Submucosal neuronal density per ganglion^a^	23 ± 1.1	22 ± 1.0	18 ± 1.1	12 ± 0.7****	14 ± 0.6	10 ± 0.4****
Submucosal ganglion area (μm^2^)^b^	4865 (3685; 6286)	5398* (4830; 6572)	3711 (2785; 5782)	4151 (3360; 5121)	4465 (3747; 5190)	4864* (4168; 5879)

^a^ Data are expressed as mean ± SEM and compared using Student's *t*‐test.

^b^ Data are expressed as median percentiles (25%; 75%) and compared using Mann–Whitney test.

* *p* < 0.05;

** *p* < 0.01;

*** *p* < 0.001;

**** *p* < 0.0001.

The neuronal density per mm^2^ of the submucosal plexus in the ileum (18%, *p* < 0.01) of the GK rats was lower than in the Wistar group. Similar to the myenteric plexus, the estimated total number of submucosal neurons was reduced in the GK group (Table 2).

**FIGURE 7:**
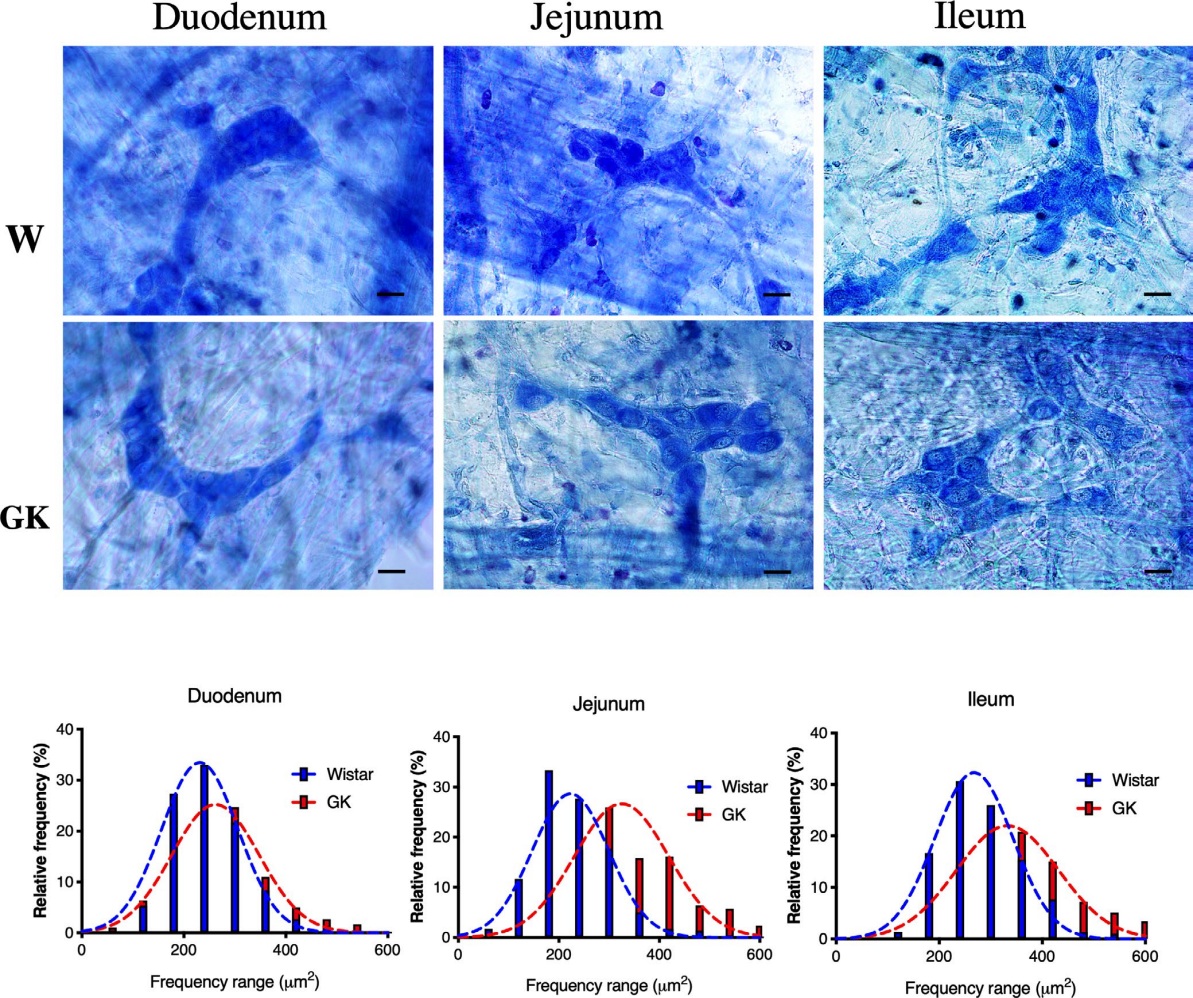
Representative photomicrographs of the submucosal neurons and frequency histogram related to the neuronal areas in the duodenum, jejunum, and ileum of the Wistar and GK groups are indicated. Objective: 40×, Scale bars: 20 μm

In the GK group, the neuronal body area was increased by 10% in the duodenum (*p* < 0.001), 72% in the jejunum (*p* < 0.0001), and 27% in the ileum (*p* < 0.0001). The neuronal body area in the duodenum ranged from 180 to 300 μm^2^ in the Wistar and GK groups. In the jejunum and ileum of the control group, the size of the neurons ranged from 180 to 300 μm^2^, whereas in the GK animals, the size of the submucosal neurons ranged from 240 to 420 μm^2^ (Table 4 and Figure 7).

### 3.11 GI transit

The GK rats exhibited slower intestinal transit. It took these animals 715 min to eliminate stained feces, while the Wistar group only took 541 min (Table 2).

## 4 DISCUSSION

In the present study, male 16‐week‐old GK animals exhibited reduced body mass, nose‐to‐tail length, and adipose tissue depots. These animals also presented fasting basal hyperglycemia, glucose intolerance, and impaired insulin sensitivity when compared to the control group. It was previously reported that at 3 weeks of age, GK rats have basal hyperglycemia, impaired insulin secretion, and increased hepatic glucose production (Picarel‐Blanchot et al., 1996; Portha et al., 2012). Additionally, at 12–16 weeks of age, the body mass is significantly low, and the increase in the white adipose tissue mass ceases. These changes are due to an impairment in pre‐adipocyte differentiation into mature adipocytes, leading to a defect in triglycerides storage and increased free fatty acid release into the plasma (Xue et al., 2011). Thus, our results are consistent with the literature and indicate that the employed experimental protocol mimics non‐obese T2DM patients (Kuwabara et al., 2017).

In response to internal and external stimuli, the intestinal adaptive capacity, known as enteroplasticity or intestinal adaptation (Drozdowski et al., 2009), occurs in short bowel syndrome, chronic alcohol ingestion, aging, fasting, malnutrition, and diabetes (Ferraris & Carey, 2000). Previous studies reported marked small intestine morphological changes in diabetes mellitus states (Zhao et al., 2006), including increased small intestinal weight and length (Zhao et al., 2003), larger mucosal surface area (Noda et al., 2001), greater goblet cell abundance (Zoubi et al., 1995), increased mucosa, submucosa, and muscular layer thickness (Zhao et al., 2003), and muscular layer hypertrophy (Nowak et al., 1990).

The results reported herein support the proposition that enteroplasticity occurs in GK rats. These animals have hyperglycemia and exhibit increased food intake that is more pronounced (relative to body mass) after weaning (Kuwabara et al., 2017; Maekawa et al., 2006). Changes in cell proliferation rate, cell number, villi length and thickness, and crypt depth have also been reported, possibly as attempts to increase the absorptive surface area and functional capacity of the intestine (Dailey, 2014). Hyperphagia occurs in several animal models of DM and can induce and/or accelerate obesity and diabetes. Indeed, data on food intake by GK animals were reported by others (Ando et al., 2018; Maekawa et al., 2006; Xue et al., 2011) and our research group (Kuwabara et al., 2017). Miller et al. (1977) investigated intestinal cell proliferation, and dry intestinal weight in pair‐fed and *ad libitum* fed diabetic animals and found that the diabetic state itself promotes marked changes in mucosal growth regardless of food intake. In this sense, although oscillations in the luminal content may affect the intestinal remodeling, the hyperglycemia presented by GK rats might be the primary cause of these changes.

Zhao et al. (2013) also reported increased villi thickness and hypertrophy of the muscular layer in the small intestine GK rat jejunum at 32 weeks of age. The authors associated the hypertrophy of the intestinal muscular layer with the enhanced proliferation of the intestinal smooth muscle cells with an inflammatory cytokine‐modulated phenotype (Nair et al., 2014).

Herein, we observed elevated levels of IL‐1β and NF‐*κ*B p65 in the small intestines of GK animals. It is known that NF‐*κ*B protein upregulates the expression of specific genes, including IL‐1β. The subsequent increase in IL‐1β activates the NF‐*κ*B pathway, promoting I*κ*B degradation and inducing NF‐*κ*B nuclear translocation. This mechanism of activation has been observed in smooth muscle and the myenteric plexus (Rumio et al., 2006). We then found a positive auto‐regulatory loop in the GK intestine that contributes to amplifying the inflammatory response and local inflammation, as previously reported (Yamamoto & Gaynor, 2001). It has also been reported that IL‐1β causes hypomotility of the intestine (Aubé et al., 1996). Therefore, it is plausible that the increase in IL‐1β and NF‐*κ*B in the small intestine contents contribute to the slower intestinal transit observed in GK rats.

Interestingly, we observed increased IL‐1β reactivity in myenteric neurons and glial cells of the small intestine of GK animals compared with the Wistar group. It has been reported that cultured myenteric glial cells secrete IL‐1β in response to LPS stimulation (Murakami et al., 2009). Under specific conditions, such as neuroinflammation, the neurons are an important source of IL‐1β and an early line of defense following injury (Rivero Vaccari et al., 2009). Myenteric cells participate in the local defense actions and inflammation by sending out signals to neighboring cells . Additionally, it has been proposed that the myenteric cells (neurons and glial cells) directly contribute to the intestinal inflammatory response by producing inflammatory mediators (Freidin et al., 1992). Therefore, it is likely that the observed increase in IL‐1β levels are due to inflammation in the small intestine of GK rats.

We did not detect a significant difference in the number of myenteric neurons per unit area or ganglion when comparing the two groups. However, diabetes has been shown to change the intestinal area and neuronal density, without any alterations in the number of neurons (Voukali et al., 2011). Herein, we observed a decrease in the total number of neurons in the myenteric plexus of GK rats, possibly due to a reduced intestinal area. In the submucosal plexus of GK rats, we found that the number of neurons in the jejunum and ileum per unit area, ganglion, and the estimated submucosal total neuronal population were all decreased. A similar reduction in submucosal total neuronal density was reported in the duodenum, jejunum, and ileum in streptozotocin‐induced diabetic animals (Ferreira et al., 2018). Our results here in GK animals show that the total neuronal population of the submucosal plexus is more susceptible to DM‐induced degenerative changes than the myenteric plexus. The degenerative changes in DM that affect the enteric nervous system are due to metabolic disorders. Furthermore, oxidative stress, resulting from the imbalance between ROS production and neutralization, is a well‐established factor that has been implicated in the pathogenesis of diabetic neuropathy and other complications (Babizhayev et al., 2015; Figueroa‐Romero et al., 2008). While our results may be related to diabetes‐induced oxidative stress, future studies investigating these mechanisms need to be conducted.

The increased neuronal body area contributed to the increased ganglion area observed in the small intestine of the GK animals. The enteric neuronal hypertrophy may represent a compensatory mechanism for maintaining the intestinal functions (Zanoni et al., 2002). Other studies reported changes in the enteric neuronal population in experimental DM models (Ferreira et al., 2013, 2018; Larsson & Voss, 2018; Lopes et al., 2012). A previous study with obese mice (ob/ob) showed that neither the density nor area of the total population in the duodenum myenteric neurons are perturbed, but differences could be observed in the myenteric neuronal‐specific subpopulations (Spangeus & El‐Salhy, 2001). Notably, in streptozotocin‐induced diabetic animals, an increase in the nitrergic neuronal population of the submucosal plexus in the jejunum and colon was reported (Bódi et al., 2017).

Moreover, reductions in the myenteric neuronal density and neuronal hypertrophy were reported in streptozotocin‐induced diabetic rats (Ferreira et al., 2018) and animals consuming a high‐fat diet (Larsson & Voss, 2018). It is important to point out that attenuated GI transit time and diarrhea were correlated with a decreased nNOS enteric inhibitory subpopulations in diabetic animals (Ferreira et al., 2018). Previous studies demonstrated that hyperglycemia‐related oxidative stress and inflammation are primary inducers of enteric nervous system dysfunction, resulting in marked changes in intestinal motility and intestinal secretion activity (Chandrasekharan et al., 2011; Trevizan et al., 2019; Voukali et al., 2011). Herein, we identified some changes in intestinal morphology and evidence of enteric nervous system remodeling in GK rats; however, the underlying mechanisms responsible for these alterations are still unclear.

It has been shown that decreased intestinal motility alters the intestinal microbiota composition, neurotransmission within the gastric wall, and gastric motility, which is regulated by afferent signals from the central nervous system (Barbara et al., 2005; Dass et al., 2007). Peng et al. (2020) reported changes in the intestinal microbiota during the aggravation of the T2DM state in GK animals, leading to higher proportions of Gram‐negative bacteria. These bacteria could trigger an inflammatory response, thus contributing to the development of the diabetic state (Larsen et al., 2010). It is important to point out that changes in intestinal microbiota composition and gut motility are concomitant processes, and it remains unclear which occurs first in the GK animal model.

There are some limitations in the present study that need to be considered when interpreting the results. We used a negative control (no primary antibody) to exclude staining artifacts in the immunohistochemistry assays, whereas a positive control was not included. Concerning the cytokine concentration measurements, we utilized complete intestinal fragments. If we had performed the measurements with separate intestinal layers (e.g., mucosa or muscular layer), additional information could have been obtained. We investigated the pan‐neuronal population of enteric plexuses in the Giemsa‐stained tissue, but enteric neuroplasticity remodeling may occur in subpopulations or enteric glia. We could have performed immunohistochemistry on the glial cells (S100), nitrergic (nNOS), and cholinergic (ChAT) neurons, which would have further strengthened the study.

In conclusion, in the present study, we identified specific alterations in the intestinal morphology and myenteric and submucosal plexuses and the presence of local inflammation in the small intestine of GK rats. These changes were accompanied by reduced intestinal transit and IR. This work provides unique insights into the molecular mechanisms underlying the onset and development of non‐obese T2DM.

## CONFLICT OF INTEREST

The authors declare that they have no competing interests.

## AUTHOR CONTRIBUTIONS

J.N.B. Pereira design, acquisition, analysis and interpretation of data, and drafting the article; G.M.M. acquisition and analysis of data; F.T.S. acquisition and analysis of data; A.R.M. analysis and interpretation of data; C.R.O.C. conception and design; R.C. conception and design, interpretation of data, drafting and paper revision.
